# Impact of prehabilitation on patient-perceived quality of recovery after surgery: prospective cohort study

**DOI:** 10.1093/bjsopen/zraf156

**Published:** 2026-01-07

**Authors:** Fernando Dana, Rubèn González-Colom, Beatriz Tena, David Capitán, Dulce Momblan, Betina Campero, Laura García Lopez, Marta Ubré, Raquel Sebio-García, Adelaida Zabalegui, Graciela Martinez-Palli, the Hospital Clinic de Barcelona Prehabilitation Group, Graciela Martínez-Pallí, Marta Ubré, Raquel Risco, Manuel López-Baamonde, Antonio López, María José Arguis, Ricard Navarro-Ripoll, Marina Sisó, Raquel Sebio, Fernando Dana, David Capitán, Amaya Peláez Sainz-Rasines, Beatriz Tena, Eva Rivas, Betina Campero, Bárbara Romano-Andrioni, Silvia Terés, Juan M Perdomo, Edgar Iglesias, María Suárez, Miguel Garriz, Maria Ona Miró

**Affiliations:** Anesthesiology Department, Hospital Clinic de Barcelona, Barcelona, Spain; Universitat de Barcelona, Barcelona, Spain; August Pi I Sunyer Biomedical Research Institute (IDIBAPS) University of Barcelona, Barcelona, Spain; Biomedical Research Networking Center on Respiratory Diseases (CIBERES), Madrid, Spain; Anesthesiology Department, Hospital Clinic de Barcelona, Barcelona, Spain; August Pi I Sunyer Biomedical Research Institute (IDIBAPS) University of Barcelona, Barcelona, Spain; Anesthesiology Department, Hospital Clinic de Barcelona, Barcelona, Spain; Universitat de Barcelona, Barcelona, Spain; Department of General and Digestive Surgery, Hospital Clinic de Barcelona, Barcelona, Spain; Department Nutrition and Clinical Dietetics, Hospital Clinic de Barcelona, Barcelona, Spain; Department of Coverage Management, Hospital Clinic de Barcelona, Barcelona, Spain; Anesthesiology Department, Hospital Clinic de Barcelona, Barcelona, Spain; August Pi I Sunyer Biomedical Research Institute (IDIBAPS) University of Barcelona, Barcelona, Spain; Physical Medicine and Rehabilitation Department, Hospital Clinic de Barcelona, Barcelona, Spain; Universitat de Barcelona, Barcelona, Spain; Subdivision of Research and Teaching in Nursing, Hospital Clinic Barcelona, Barcelona, Spain; Anesthesiology Department, Hospital Clinic de Barcelona, Barcelona, Spain; August Pi I Sunyer Biomedical Research Institute (IDIBAPS) University of Barcelona, Barcelona, Spain; Biomedical Research Networking Center on Respiratory Diseases (CIBERES), Madrid, Spain

**Keywords:** gastrointestinal surgery, multimodal prehabilitation, patient-reported outcome measures, postoperative recovery, preoperative care, QoR-15

## Abstract

**Background:**

Multimodal prehabilitation has the potential to reduce complications, shorten hospital stays, and decrease healthcare resource utilization. However, its impact on patient-centred outcomes, such as patient reported-outcomes, has been less extensively studied. This study assessed the effect of multimodal prehabilitation on patient-perceived quality of recovery following elective surgery.

**Methods:**

This was a prospective cohort study of patients undergoing elective gastrointestinal surgery between 1 February 2024 and 28 February 2025 who met institutional criteria for prehabilitation. Outcomes, including comparing postoperative complications, length of hospital stay, and perceived recovery, were compared between patients who completed the prehabilitation program and those who did not (control cohort). The primary outcome measure was the Quality of Recovery-15 (QoR-15) questionnaire score.

**Results:**

In all, 188 patients were included in the study. The 94 patients who completed the prehabilitation program over a mean(standard deviation) of 4.5(1.6) weeks had fewer postoperative complications per patient than did patients in the control group (mean(standard deviation) 1.0(1.4) *versus* 1.4(1.4); *P* = 0.008). In addition, mean(standard deviation) QoR-15 scores were significantly higher in the prehabilitation than control group at baseline (129.5(15.0) *versus* 122.9(17.0); *P* = 0.003), discharge (117.2(14.0) *versus* 106.8(15.0); *P* < 0.001), and 30 days after discharge (128.2(16.0) *versus* 118.5(14.0); *P* < 0.001). At 30 days after discharge, 66% of patients in the prehabilitation group had recovered all three pre-identified essential activities, compared with 35% in the control group (*P* = 0.001).

**Conclusions:**

The findings suggest that prehabilitation not only reduces postoperative morbidity and facilitates physical recovery but also enhances patients’ subjective experience of recovery throughout the surgical journey, supporting its integration into routine perioperative care for digestive surgery.

## Teaser

This prospective cohort study evaluated the impact of multimodal prehabilitation on patient-perceived recovery after digestive surgery. Patients undergoing prehabilitation had significantly higher Quality of Recovery-15 questionnaire scores and fewer postoperative complications. The findings suggest that prehabilitation improves both clinical outcomes and the overall recovery experience, even in patients who experience postoperative complications.

## Introduction

Healthcare quality has traditionally been assessed through clinical outcomes such as treatment response, complications rates, or survival. However, patients’ priorities often differ from those of healthcare professionals^1,2^. Since 2008, the Institute for Healthcare Improvement has proposed the Triple Aim framework^3^ as an innovative approach to optimizing healthcare systems through the simultaneous pursuit of three interrelated objectives: improving the patient experience (including quality and satisfaction), enhancing population health, and reducing per-capita healthcare costs.

This approach has evolved into the Quintuple Aim^4^, which integrates provider wellbeing and health equity as additional interconnected objectives. This fosters a more equitable, effective, and sustainable healthcare system. The assessment of patient experience within healthcare interventions has become an integral part of evaluating healthcare quality, aligning with the principles of patient-centred care^5–7^. Patients’ perspectives about their healthcare experience can play a crucial role in hospital management^8^; consequently, patient-reported outcome and experience measures (PROMs and PREMs, respectively) have gained significant traction as integrated tools to evaluate healthcare goals among patients and healthcare users^9–11^. In the surgical setting, where surgery is often part of a broader process, the Quality of Recovery 15-item questionnaire (QoR-15)^12^ has gained popularity for its accurate assessment following surgery, enabling precise evaluation of recovery from the patient’s perspective and supporting both personalized care and shared clinical decision-making.

Prehabilitation is defined as a patient-tailored preoperative intervention encompassing exercise training, physical activity, nutritional optimization, and psychological support^13,14^. Multimodal prehabilitation programs have been shown to be effective in improving preoperative functional capacity, minimizing postoperative morbidity, and accelerating recovery^15,16^. Numerous studies have demonstrated that prehabilitation helps reduce hospital stays and postoperative complications, especially in high-risk patients or those undergoing complex surgeries^17^. However, despite these well documented clinical benefits^18,19^, the impact of prehabilitation on PREMs and PROMs has received significantly less attention. It remains unclear whether the reduction in postoperative morbidity is accompanied by improved patient-perceived care and recovery. As patient experience is becoming increasingly relevant in the evaluation of healthcare interventions, it is imperative to determine whether prehabilitation programs enhance self-perceived recovery after surgery.

The main objective of this study was to compare patient-perceived recovery from surgery at hospital discharge between two cohorts: one that participated in a multimodal prehabilitation program and a contemporaneous cohort that did not receive the intervention. Secondary objectives included assessing differences between the two cohorts in postoperative complications, length of hospital stay, and recovery of daily activities 1 month after discharge.

## Methods

### Study design

A prospective cohort study was conducted to evaluate the impact of a multimodal prehabilitation program on patients’ quality of recovery at a tertiary care hospital (Hospital Clinic of Barcelona) between 1 February 2024 and 28 February 2025. The study was approved by the local ethics committee (Approval no. HCB/2023/0941) and all patients provided written informed consent before enrolment. The Strengthening the Reporting of Observational Studies in Epidemiology (STROBE)^20^ guidelines were followed in reporting this study (*Supplementary material*).

Patients scheduled for major elective gastrointestinal surgery who fulfilled the following institutional criteria were considered for prehabilitation: moderate to high risk of postoperative complications, defined as American Society of Anesthesiologists (ASA)^21^ grade III or IV; and/or age ≥ 70 years; and/or the presence of cancer-related deconditioning due to disease and/or treatment; and/or patients undergoing highly complex surgeries according to the National Institute for Health Care Excellence (2016)^22^ classification.

Patients who declined participation in the prehabilitation program or those referred less than 2 weeks before surgery (due to logistical or organizational constraints, rather than clinical criteria) were assigned to the control cohort. Patients with neurological or cognitive impairment or language barriers preventing them from understanding and completing the QoR-15 questionnaire were excluded from the study.

### Intervention: multimodal prehabilitation program

The multimodal prehabilitation program was delivered by a multidisciplinary team comprising anaesthesiologists, physiotherapists, dieticians, psychologists, and nurses. Full details of the program can be found elsewhere^17^. Briefly, the intervention consisted of the following:

- Functional optimization: patients participated in supervised aerobic and resistance training sessions two to three times per week, each session lasting approximately 60 minutes. Exercise intensity was individually tailored according to patients’ baseline functional capacity and was increased weekly as tolerated. In addition, a goal-setting physical activity program was provided. Patients were encouraged to increase their daily step count by 500–1000 steps/day each week, if feasible, and to minimize sedentary behaviour. Breathing exercises were also introduced if patients were scheduled for open surgery or considered at increased risk of postoperative pulmonary complications.
- Nutritional intervention: nutritional status was assessed using the Malnutrition Universal Screening Tool^23^ and malnutrition was diagnosed according to the Global Leadership Initiative on Malnutrition^24^ criteria. All patients received dietary counselling focused on protein enrichment to support increased physical activity. In patients diagnosed with malnutrition, nutritional supplementation was prescribed to ensure adequate caloric and protein intake through diet therapy and supplementation. In addition, these patients were followed up fortnightly with telephone calls.
- Psychological support: patients completed the Hospital Anxiety and Depression Scale (HADS)^25^ questionnaire and were offered an individualized consultation with the unit’s psychologist; particular emphasis was placed on those presenting with higher emotional distress (HADS scores ≥ 8), who were referred for a more thorough assessment and individualized follow-up.

Patients who did not participate in the prehabilitation program received standard preoperative care, including general recommendations on physical activity, nutrition, smoking cessation, and alcohol reduction, from their anaesthetist. Patients diagnosed with iron-deficiency anaemia were treated with intravenous iron. Both the control and prehabilitation cohorts followed enhanced recovery after surgery^26^ protocols.

### Outcome measures

The primary outcome of this study was patient-perceived postoperative recovery at hospital discharge, measured using the validated Spanish version of the QoR-15^27^. This instrument has demonstrated strong internal consistency in the postoperative period (Cronbach's alpha = 0.856) and excellent test–retest reliability (intraclass correlation coefficient = 0.998; 95% confidence interval 0.996 to 0.999). The questionnaire includes 15 items, each rated from 0 to 10, with total scores ranging from 0 to 150 and higher scores indicating better recovery. QoR-15 scores were categorized^28^ as excellent (> 135), good (122–135), moderate (90–121), or poor (< 90). According to current literature^29^, the minimal clinically important difference (MCID) for the QoR-15 has been identified as 6 points.

In addition, at the time of study inclusion, patients were asked to identify, using a customized questionnaire, three activities of daily living that they consider essential to resume at 30 days after hospital discharge. At 30 days after surgery, using the same questionnaire, patients were asked to identify which of these three activities they had recovered. To this end, the questionnaire included the following open-ended question: ‘Which activities are the most important to you that you would like to resume after surgery?’. Participants were allowed to list up to three activities, listing one, two or three activities in no particular order.

Patients were assessed at three time points: before surgery at study inclusion (T0; within 24 hours before surgery), the day of hospital discharge (TD), and 30 days after hospital discharge (T30). The questionnaire was administered by a member of the research team who was blinded to the patient's cohort allocation. Similarly, hospital discharge was determined by the attending surgical team in accordance with standardized institutional care criteria.

Demographic and clinical data collected included age, sex, ASA classification, frailty status (evaluated using the Canadian Health and Safety Assessment Clinical Frailty Scale^30^), neoadjuvant therapy, surgical approach (open *versus* laparoscopic/robotic), and duration of surgery (in minutes). Postoperative outcomes included complications graded according to the Clavien–Dindo classification^31^, the Comprehensive Complication Index^32^, length of hospital stay (LOS), intensive care unit (ICU) admissions, and ICU length of stay. Emergency department visits and hospital readmissions within 30 days after discharge were also recorded.

In addition, for the prehabilitation cohort, program adherence to exercise training (planned *versus* completed sessions) was recorded by the clinical team, along with program duration and any adverse events. Functional capacity was assessed both in the preoperative period and at 30 days after discharge using the six-minute walk test (6MWT)^33^ and the 30-second sit-to-stand test (30STS)^34^. In addition, physical activity levels were recorded using the self-reported Yale Physical Activity Survey^35^ (YPAS) questionnaire.

### Statistical analysis and sample size calculation

Sample size estimation was based on previously published data reporting a common standard deviation of the QoR-15 score of 17.1 points^28^. To detect a 6-point^30^ MCID with 80% power and a 5% type I error, a total sample of 180 patients was required, including a 10% dropout rate.

Baseline characteristics between the study cohorts were compared using appropriate univariate statistical tests based on variable type and distribution. Continuous variables were analysed using Student’s *t* test or the Wilcoxon rank-sum test, whereas categorical variables were compared using χ^2^ or Fisher’s exact tests, as appropriate. Variables showing baseline imbalances were included as covariates in the subsequent models.

Recovery trajectories were analysed using the QoR-15 scale through a linear mixed-effects (LME) model. The primary outcome was assessed at three time points: T0, TD, and T30. Time, cohort allocation (control *versus* prehabilitation), and their interaction were specified as fixed effects. Patient-level random intercepts were included to account for repeated measurements within individuals. The imbalanced baseline variables between study groups were subsequently included as covariates in the linear mixed-effects model to adjust for potential confounding; these included ASA classification, frailty status, education level, and receipt of preoperative neoadjuvant therapy. Estimated marginal means were calculated, and pairwise comparisons between groups and time points were performed with Bonferroni adjustment to control for multiple testing.

Throughout the paper, continuous variables are reported as the mean with standard deviation (s.d.) or median and interquartile range, depending on their distribution, whereas categorical variables are presented as absolute and relative frequencies. *P* < 0.05 was considered significant. Analyses were performed using SPSS^®^ version 26 for Windows^©^ (IBM, Armonk, NY, USA) and R version 4.1.0 (R Foundation for Statistical Computing, Vienna, Austria).

## Results

**Fig. 1:**
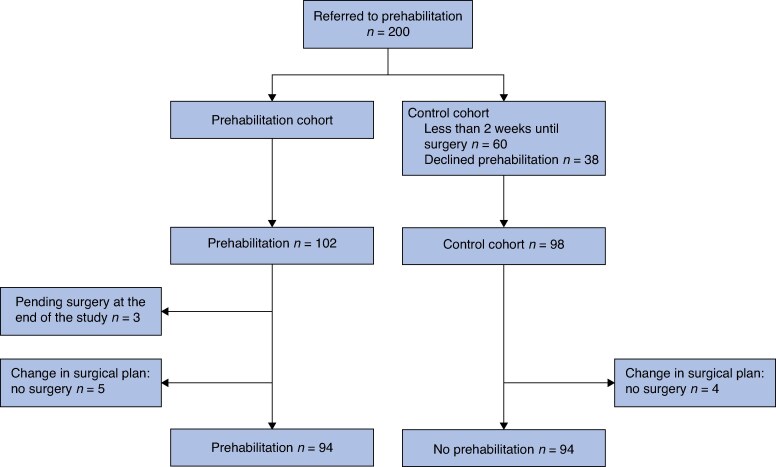
Flowchart of study enrolment

Of the 200 patients referred to the unit, 102 engaged in the prehabilitation program. Of these, five patients did not undergo surgery, and three had not undergone surgery by the end of the study period (*Fig. 1*). The control cohort consisted of 98 patients. Of these, 38 actively declined participation in the prehabilitation program. Reported reasons included logistical difficulties to attend (22 patients), such as a lack of support, transportation issues, or scheduling conflicts, or simply a lack of motivation to engage in the exercise program (16 patients). Four patients were not operated on and were therefore excluded from the analysis. The final analysis included 94 patients in each cohort.

Baseline characteristics for both cohorts are presented in *Table 1*. Initial comparisons revealed no differences between cohorts in terms of age or sex distribution (*P* = 0.059). Patients in the prehabilitation cohort had higher ASA grades (*P* = 0.029), greater frailty (*P* = 0.009) and lower educational attainment (*P* < 0.001), and a higher proportion of patients in the prehabilitation cohort received neoadjuvant therapy (*P* = 0.007). No significant differences were observed between cohorts regarding the type of surgery performed (*P* = 0.321).

**Table 1:** Preoperative clinical data and type of surgery by group

	Prehabilitation (*n* = 94)	Control (*n* = 94)	P*
Age (years), mean(s.d.)	71.5(10.3)	70.0(12.7)	0.666
**Sex**			0.059
Male	58 (62%)	44 (47%)	
Female	36 (38%)	50 (53%)	
ASA grade ≥ III	68 (72%)	51 (54%)	0.029
CHSA† CFS ≥ 4	45 (48%)	36 (38%)	0.009
**Educational attainment**			< 0.001
None	1 (1%)	4 (4%)	
Primary	46 (49%)	21 (23%)	
Secondary	31 (33%)	24 (26%)	
University	13 (14%)	27 (29%)	
Ph.D.	2 (2%)	4 (4%)	
Professional education	1 (1%)	13 (14%)	
Neoadjuvant therapy	37 (39%)	18 (19%)	0.007
**Type of surgery**			0.321
Colorectal resection	34 (36%)	34 (36%)	
Liver resection	6 (6%)	7 (7%)	
Bariatric	2 (2%)	3 (3%)	
Gastrectomy	8 (9%)	7 (7%)	
Oesophagectomy	19 (20%)	8 (9%)	
Pancreatectomy	8 (9%)	15 (16%)	
HIPEC	9 (10%)	13 (14%)	
Other‡	8 (9%)	7 (7%)	
**Surgical approach**			0.982
Open	21 (22%)	24 (26%)	
Laparoscopic	38 (40%)	36 (3%)	
Robotic	35 (37%)	34 (3%)	
Surgical time (min), mean(s.d.)	282.7(149.6)	282.6(162.2)	0.900

Values are *n* (%) unless otherwise stated. †The CHSA CFS classifies patients into nine levels based on their functional status and degree of dependency from ‘very fit’ (1) to ‘terminally ill’ (9). Patients scoring ≥ 4 can be considered vulnerable. ‡Other includes complex abdominal wall surgery and supra-adrenalectomy. All procedures included were classified as major surgeries according to the criteria of the National Institute for Health and Care Excellence. s.d., standard deviation; ASA, American Society of Anesthesiologists; CHSA CFS, Canadian Health and Safety Assessment Clinical Frailty Scale; HIPEC, hyperthermic intraperitoneal chemotherapy; min, minutes. Between-group comparisons for numerical variables were performed using two-tailed Student's t-tests or Wilcoxon rank-sum tests according to parametric assumptions. Categorical variables were analysed using Chi-square or Fisher's exact tests based on expected frequencies. Statistical significance was set at *P* < 0.05.

### Effect of prehabilitation on patient experience

**Fig. 2:**
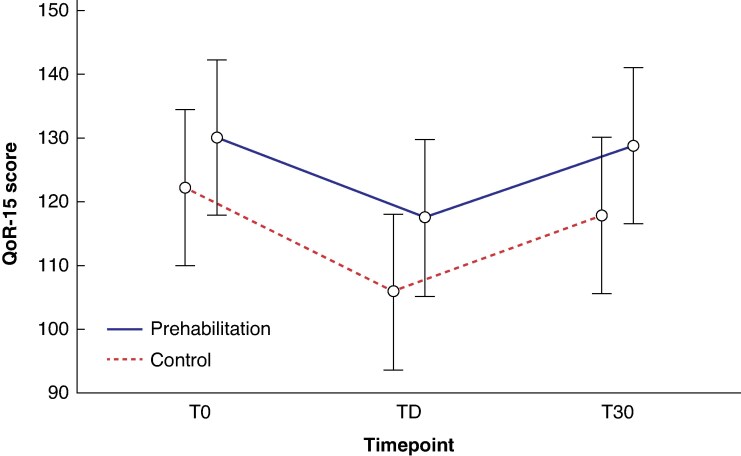
Estimated marginal means of QoR-15 scores across study cohorts and time points Mean estimated marginal means of QoR-15 scores at baseline (T0), TD, and T30 for the prehabilitation and control cohorts. The prehabilitation cohort consistently showed higher recovery scores across all time points. Error bars represent the standard deviation. QoR-15, Quality of Recovery 15-item questionnaire; T0, before surgery; TD, discharge day; T30, 30 days after surgery.

*Table 2* presents QoR-15 scores in the prehabilitation and control cohorts at each of the three time points (T0, TD, and T30), including both continuous scores and categorical classifications. In addition, *Fig. 2* shows estimated marginal means derived from the linear mixed-effects models, which account for repeated measures and adjust for potential confounding variables such as ASA grade, frailty status, educational level, and neoadjuvant therapy. Both approaches yielded concordant results and are described in detail below.

**Table 2:** QoR-15 scores at different time points

	Prehabilitation (*n* = 94)	Control (*n* = 94)	P*
Baseline QoR-15 score, mean(s.d.)	129.5(15.0)	122.9(17.0)	0.003
**Baseline QoR-15 category**			0.001
Excellent	42 (45%)	18 (20%)	
Good	22 (23%)	44 (47%)	
Moderate	29 (31%)	26 (28%)	
Poor	1 (1%)	5 (5%)	
Discharge QoR-15 score, mean(s.d.)	117.2(14.0)	106.8(15.0)	< 0.001
**Discharge QoR-15 category**			< 0.001
Excellent	4 (4%)	2 (2%)	
Good	35 (38%)	10 (11%)	
Moderate	52 (55%)	70 (74%)	
Poor	3 (3%)	12 (13%)	
30-day QoR-15 score, mean(s.d.)	128.2(16.0)	118.5(14.0)	< 0.001
**30-day QoR-15 category**			< 0.001
Excellent	34 (37%)	8 (9%)	
Good	31 (34%)	42 (45%)	
Moderate	27 (29%)	40 (43%)	
Poor	0	3 (3%)	

Values are *n* (%) unless otherwise stated. Patients were divided into recovery categories based on QoR-15 scores as follows: excellent, score ≥ 136; good, score 122–135; moderate, score 90–121; and poor, score < 90. QoR-15, Quality of Recovery 15-item questionnaire; s.d., standard deviation. *Between-group comparisons for numerical variables were performed using two-tailed Student's t-tests or Wilcoxon rank-sum tests according to parametric assumptions. Categorical variables were analysed using Chi-square or Fisher's exact tests based on expected frequencies. Statistical significance was set at *P* < 0.05.

Patients who participated in the prehabilitation program had significantly higher QoR-15 scores at all time points evaluated. Post hoc comparisons confirmed a consistent advantage for the prehabilitation cohort at T0 (*P* = 0.003), TD (*P* < 0.001), and T30 (*P* < 0.001) compared with the control group. The greatest benefit was observed at discharge, with a mean(s.d.) difference of 10.6(2.1) points (*Table 2*).

No significant interactions between group and time were detected at discharge (*P* = 0.436) or at 30 days (*P* = 0.166), indicating that both groups exhibited a similar temporal change in QoR-15 scores after surgery. Within-group temporal contrasts showed that in both groups QoR-15 scores worsened significantly from T0 to TD (*P* < 0.001) and partially improved from TD to T30 (*P* < 0.001). However, full recovery to baseline levels was only observed in the prehabilitation cohort, with no significant difference between T0 and T30 (*P* = 1.000); in contrast, the control cohort showed a residual deficit at T30 (*P* = 0.046).

**Fig. 3:**
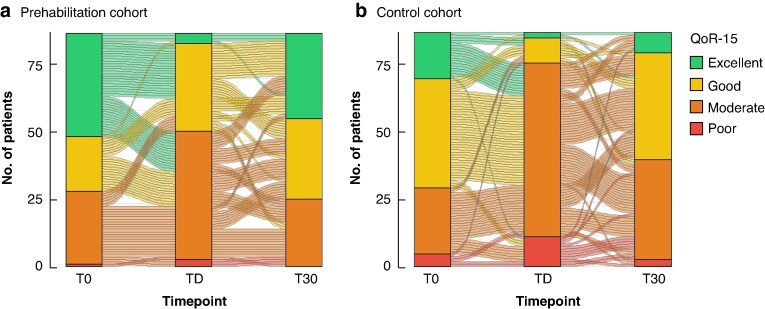
Sankey diagram showing changes in QoR-15 scores over time The diagram shows the changes in the number of patients in each QoR-15 category (excellent, good, moderate, poor) from baseline (T0) to TD and T30 in the **a** prehabilitation and **b** control cohorts. Patients in the prehabilitation group maintained a more stable distribution across categories over time, with fewer patients shifting towards worse QoR-15 categories at TD and a greater proportion returning to higher categories by T30, compared with the control cohort. QoR-15, Quality of Recovery 15-item questionnaire; T0, before surgery; TD, discharge day; T30, 30 days after surgery.

**Fig. 4:**
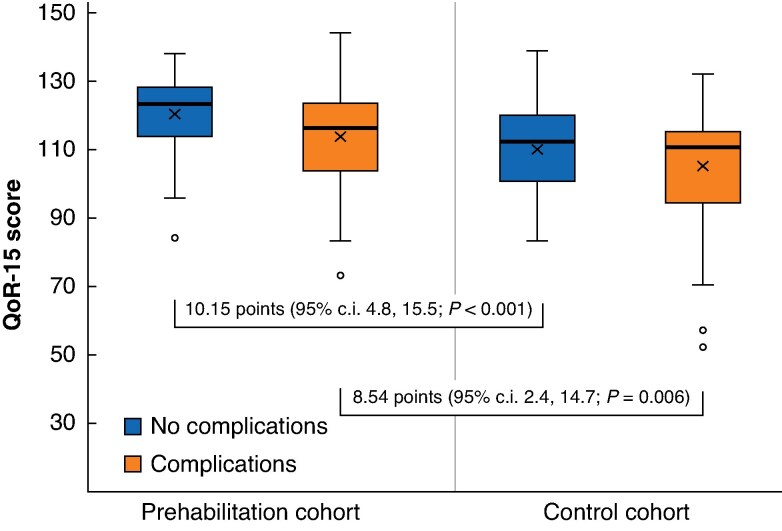
QoR-15 scores at hospital discharge by cohort and complication status QoR-15 scores in patients in the prehabilitation and control cohorts, with and without complications. The boxes show the interquartile range, with the median value indicated by the horizontal line; whiskers show the range. The mean (standard deviation) QoR-15 scores (‘X’) were 113.5 (15.1) and 120.2 (11.1) in the prehabilitation cohort with and without complications, respectively, and 105.0 (15.9) and 110.0 (13.0) in the control cohort with and without complications, respectively. QoR-15, Quality of Recovery15-item questionnaire; c.i., confidence interval.

*Figure 3* shows changes in QoR-15 categories across the three time points (T0, TD, and T30) separately for the control and prehabilitation cohorts. In addition, QoR-15 scores were higher in patients in the prehabilitation cohort than in the control group regardless of the incidence of postoperative complications (*Fig. 4*).

Finally, 30 days after hospital discharge, 57 patients (66%) in the prehabilitation cohort reported having recovered the three activities they had identified as essential to them, compared with only 29 patients (35%) in the control cohort (*P* = 0.001; *Table 3*).

**Table 3:** Postoperative outcomes

	Prehabilitation (*n* = 94)	Control (*n* = 94)	P*
Patients with no complications during admission	49 (52%)	34 (36%)	< 0.001
Total no. of complications, mean(s.d.)	1.0(1.4)	1.4(1.4)	0.008
CCI, mean(s.d.)	11.9(16)	14.9(15)	0.290
LOS (days), mean(s.d.)	8.7(9)	9.2(12)	0.622
ICU admission	45 (48%)	41 (44%)	0.526
ICU† (days), mean(s.d.)	3(4.6)	2.5(2.8)	0.518
**Patient destination at hospital discharge**			0.003
Discharge to home	76 (83%)	67 (71%)	
Discharge to home with support services	10 (11%)	25 (29%)	
Convalescent care facility	6 (6%)	1 (1)	
Post-discharge emergency visit	16 (17%)	27 (29%)	0.071
Post-discharge readmission	11 (12%)	14 (16%)	0.367
**No. of essential activities recovered 30 days after discharge‡**			0.001
0	6 (6%)	11 (1%)	
1	9 (10%)	25 (30%)	
2	20 (23%)	28 (34%)	
3	57 (66%)	29 (35%)	

Values are *n* (%) unless otherwise stated. †The number of ICU days was calculated only for patients admitted to the ICU. ‡Before undergoing surgery, patients specified three activities they considered essential to recover by 30 days after discharge. s.d., standard deviation; CCI, Comprehensive Complication Index; LOS, length of hospital stay; ICU, intensive care unit. *Between-group comparisons for numerical variables were performed using two-tailed Student's t-tests or Wilcoxon rank-sum tests according to parametric assumptions. Categorical variables were analysed using Chi-square or Fisher's exact tests based on expected frequencies. Statistical significance was set at *P* < 0.05.

### Impact of prehabilitation on surgical outcomes

The incidence of postoperative complications per patient was lower in the prehabilitation than control group (1.0 *versus* 1.4 per patient, respectively; *P* = 0.008). There were no statistically significant differences between the two groups in length of hospital stay, ICU admissions, or ICU length of stay (*Table 3*). A greater proportion of patients were reported to need a caregiver after hospital discharge in the control compared with prehabilitation cohort (29 *versus* 11%, respectively; *P* = 0.003).

### Impact of prehabilitation in functional status

Prehabilitation patients completed a mean(s.d.) of 86(15)% of scheduled exercise sessions over a mean(s.d.) of 4.5(1.6) weeks. Significant improvements were observed between the baseline and presurgical assessment in functional capacity, with mean(s.d.) changes in the 6MWT of 14.4(47.4) m (*P* = 0.023), 30STS of +2.3(3) repetitions (*P* < 0.001), and YPAS of +18.4(14.7) (*P* < 0.001). At 30 days after discharge, patients had declined to baseline values in the 6MWT (mean(s.d.) difference −1.5(53.4) m; *P* = 0.800), showed a sustained improvement in the 30STS (+1.1(2.5) repetitions; *P* = 0.002), and did not show a significant improvement in YPAS scores (+2.3(12.7); *P* = 0.135).

### Nutritional and psychological follow-up

All patients included in the prehabilitation program underwent an individual nutritional assessment at both the beginning and end of the intervention. According to Global Leadership Initiative on Malnutrition criteria, 13 patients were diagnosed with severe malnutrition and 28 were diagnosed with moderate malnutrition. During the course of the program, 10 patients attended an additional in-person visit, whereas 47 were contacted by telephone to monitor their progress. Furthermore, 32 patients received an immunomodulatory formula during the week before surgery as part of the nutritional optimization strategy.

Among the patients included in the prehabilitation program, 44 (47%) had HADS scores > 8. Of these patients, 26 (59%) completed individualized weekly follow-up, 2 (5%) were referred to specialized psychological services, and the remaining 16 (36%) declined psychological intervention.

## Discussion

This study specifically evaluated the impact of prehabilitation on patient-perceived postoperative recovery at the time of hospital discharge. The results suggest that, in a real-world clinical setting involving major gastrointestinal surgery, prehabilitation may be associated with improved surgical outcomes and a more positive overall patient experience, regardless of the incidence of postoperative complications. In addition, patients who underwent prehabilitation regained a greater proportion of their key daily activities within 30 days of discharge, which was reflected in the recovery of their baseline functional capacity. These findings support the potential value of multimodal prehabilitation in the surgical setting, both in terms of postoperative outcomes and in improving patient experience and recovery of autonomy after major surgery.

Prehabilitation has been associated with favourable surgical outcomes, such as reduced postoperative complications, shorter LOS, or enhanced postoperative functional recovery^36,37^. Consistent with these findings, patients in the prehabilitation cohort in the present study experienced fewer postoperative complications than those in the control group (1.0 *versus* 1.4; *P* = 0.008), with a trend towards shorter LOS. However, there is limited evidence in the current literature regarding the potential impact of prehabilitation on patients’ perception of recovery following surgery. The scarce data available are from one study conducted in a population undergoing cardiac surgery^38^ and another involving patients undergoing colorectal surgery^39^. In both studies, recovery perception was assessed using the QoR-15, which has been extensively used in surgical populations^40,41^ and has demonstrated sensitivity to changes in patients’ health status^42^. This makes it a useful PROM, because its composite recovery score allows for meaningful comparisons between interventions^43^. Moreover, the availability of an established MCID enhances its applicability in clinical trials and outcome studies^44^.

The studies referenced above^38,39^ yielded contradictory results. Specifically, prehabilitation demonstrated positive effects on quality of recovery in patients undergoing colorectal surgery at 72 hours after surgery^39^, but not in those undergoing cardiac surgery^38^. This discrepancy may stem from the observation that the beneficial outcomes of prehabilitation in patients undergoing colorectal surgery have not been robustly reproduced in the cardiac surgery population^45^. It is likely that surgical patients will exhibit varying responses to this therapeutic strategy. In addition, the discrepancy may be due to fact that the timing of the QoR-15 assessment is arbitrary. The decision to measure QoR-15 at hospital discharge in the present study was motivated by the heterogeneity of surgical procedures included, which led to diverse recovery patterns. The aim of this study was to capture the patient’s perception at the moment clinicians consider hospital treatment no longer necessary, providing a consistent benchmark across patients.

The observed difference in QoR-15 scores at hospital discharge (10.4 points) between cohorts exceeded the established MCID of 6 points, suggesting that prehabilitation may meaningfully improve the perception of postoperative recovery in patients undergoing major gastrointestinal surgery. The fact that patients who engaged in prehabilitation reported higher QoR-15 scores before surgery, after completing the program, supports existing evidence that prehabilitation enhances physical and psychological wellbeing, as reported in previous randomized clinical trials^46–48^. The QoR-15 assesses key domains such as pain, physical comfort, functional independence, psychological support, and emotional state, all of which are directly influenced by multimodal prehabilitation interventions. Notably, this difference persisted over time, suggesting that the relative advantage associated with prehabilitation was maintained consistently throughout the postoperative course. A QoR-15 score of ≥ 118 has been documented to reflect good postoperative recovery^42^. Bearing this in mind, the mean QoR-15 score at hospital discharge was higher in the prehabilitation cohort (117.0 points) than in the control cohort (106.0 points; *P* < 0.001). A significantly greater proportion of patients in the prehabilitation than control group achieved or exceeded the 118-point threshold indicative of good recovery (51 *versus* 21%, respectively), reinforcing the clinical relevance of the observed improvement.

It may be argued that the difference in quality of recovery is simply due to prehabilitated patients experiencing fewer postoperative complications during their hospital stay. However, prehabilitated patients still exhibited a better quality of recovery than control patients, even when their postoperative course was complicated (*P* = 0.006). This is consistent with the findings of Ten Cate *et al*.^49^ in a multicentre randomized trial in colorectal surgery, who found that regardless of postoperative complications, patients undergoing prehabilitation exhibited faster recovery of functional capacity measured by 6MWT.

In the present study, the improvement in perceived recovery was accompanied by a greater extent of recovery of preferred activities by patients in the prehabilitation compared with control cohort. In particular, 57 patients (66%) in the prehabilitation cohort reported having resumed all three of their prioritized activities by 30 days after discharge, compared with 29 patients (35%) in the control cohort (*P* = 0.0001). This suggests that prehabilitation supports not only medical recovery but also a return to patients’ normal routines and home environments.

This study has several limitations. First, the prospective cohort study design is not optimal for comparing groups on the primary outcome. Notably, the control cohort included patients who were less frail and had fewer co-morbidities. Although this may highlight the beneficial effects of prehabilitation in high-risk patients, it introduces a selection bias. This reflects real-world clinical practice, where patients perceived as more vulnerable by the surgeon are referred earlier to the prehabilitation unit or have their surgery postponed until they complete the program. To address this, adjustments were made for key baseline differences (ASA grade, frailty status, education level, and neoadjuvant therapy) in the statistical models. Although this does not eliminate the bias entirely, it mitigates its impact. The observational design was chosen because prehabilitation is already standard of care in the study hospital, and it was not ethically appropriate to deny access to patients who could benefit from it. This methodological decision allowed evaluation of the impact of the intervention in a real-world clinical setting, aligned with the principles of pragmatic research, which aim to generate evidence applicable to routine practice. Patients involved in prehabilitation showed an improvement in functional capacity that may explain the better perception of their health status. In addition, the study was conducted at a single institution, which limits its external validity. Finally, patients in the control cohort did not undergo all assessments (for example functional capacity), which restricts comparisons in some domains.

## Collaborators

The members of the Hospital Clinic de Barcelona Prehabilitation Group are: Graciela Martínez-Pallí, Marta Ubré, Raquel Risco, Manuel López-Baamonde, Antonio López, María José Arguis, Ricard Navarro-Ripoll, Marina Sisó, Raquel Sebio, Fernando Dana, David Capitán, Amaya Peláez Sainz-Rasines, Beatriz Tena, Eva Rivas, Betina Campero, Bárbara Romano-Andrioni, Silvia Terés, Juan M Perdomo, Edgar Iglesias, María Suárez, Miguel Garriz, and Maria Ona Miró. All collaborators are affiliated with the Prehabilitation Unit, Department of Anesthesiology, Hospital Clínic de Barcelona, Barcelona, Spain.

## Supplementary Material

zraf156_Supplementary_Data

## Data Availability

The data sets generated and analysed during this study are not publicly available due to ethical and legal restrictions under applicable data protection regulations. However, data may be available upon reasonable request to the corresponding author, subject to approval by the relevant ethics committees.
