# Supplementary material for: Impact of prehabilitation on patient-perceived quality of recovery after surgery: prospective cohort study
Source: BJS Open. 2026 Jan 7;10(1):zraf156. doi: 10.1093/bjsopen/zraf156 (PMC12777966; doi:10.1093/bjsopen/zraf156)
Supplement: zraf156_Supplementary_Data [file zraf156_supplementary_data.docx]

**Impact of prehabilitation on patient-perceived quality of recovery after surgery: prospective cohort study**

**Authors:** Fernando Dana^1,5^, Rubèn González-Colom^2,4^, Beatriz Tena^1,2^, David Capitan^1^, Dulce Momblan^5,6^, Betina Campero^8^, Laura García Lopez^9^, Marta Ubré^1^, Raquel Sebio-García^2,3*^, Adelaida Zabalegui^5,7†^, G. Martínez-Pallí^1,2,4†^, on behalf of the Hospital Clinic de Barcelona Prehabilitation Group (+).

*Corresponding author

†Adelaida Zabalegui and G. Martínez-Pallí have equally contributed to this work and shared last co-authorship.

(+) Hospital Clinic de Barcelona Prehabilitation Group Collaborators: Graciela Martínez-Pallí, Marta Ubré, Raquel Risco, Manuel López-Baamonde, Antonio López, María José Arguis, Ricard Navarro-Ripoll, Marina Sisó, Raquel Sebio, Fernando Dana, David Capitán, Amaya Peláez Sainz-Rasines, Beatriz Tena, Eva Rivas, Betina Campero, Bárbara Romano-Andrioni, Silvia Terés, Juan M Perdomo, Edgar Iglesias, María Suárez, Miguel Garriz and Maria Ona Miró.

**Affiliations:**

^1^Anesthesiology Department, Hospital Clinic de Barcelona, Barcelona, Spain.

^2^August Pi i Sunyer Biomedical Research Institute (IDIBAPS) University of Barcelona, Barcelona, Spain.

^3^Physical Medicine and Rehabilitation Department, Hospital Clinic de Barcelona, Barcelona, Spain.

^4^Biomedical Research Networking Center on Respiratory Diseases (CIBERES), Madrid, Spain.

^5^Universitat de Barcelona, Barcelona, Spain.

^6^Department of General and Digestive Surgery, Hospital Clinic de Barcelona, Barcelona, Spain.

^7^Subdivision of Research and Teaching in Nursing, Hospital Clinic Barcelona, Barcelona, Spain.

^8^Department Nutrition and Clinical Dietetics, Hospital Clinic de Barcelona, Barcelona, Spain.

^9^Department of Coverage Management, Hospital Clinic de Barcelona, Barcelona, Spain.

# Corresponding author*

Raquel Sebio-García

Email: sebio@clinic.cat

**Supplementary Materials - Index**

| **Supplementary Methods** |  |
| --- | --- |
| STROBE Statement | *page 4* |
|  |  |

**Supplementary Methods**

STROBE Statement—Checklist of items that should be included in reports of ***cohort studies***

|  | Item No | Recommendation |
| --- | --- | --- |
| Title and abstract | 1 | (*a*) Indicate the study’s design with a commonly used term in the title or the abstract  A prospective cohort study, as stated in the Methods section of the abstract and in the Study Design section of the Methods. Page 3. |
|  |  | (*b*) Provide in the abstract an informative and balanced summary of what was done and what was found  It is presented in the study's abstract. Page 3. |
| Introduction | | |
| Background/rationale | 2 | Explain the scientific background and rationale for the investigation being reported  Included in the Introduction, first paragraph. Page 4 |
| Objectives | 3 | State specific objectives, including any prespecified hypotheses  Included in the Introduction, last paragraph. Page 5 |
| Methods | | |
| Study design | 4 | Present key elements of study design early in the paper  Included in the study design subsection within the Materials and Methods section. Page 6. |
| Setting | 5 | Describe the setting, locations, and relevant dates, including periods of recruitment, exposure, follow-up, and data collection  Included in the study design subsection within the Materials and Methods section. Page 6. |
| Participants | 6 | (*a*) Give the eligibility criteria, and the sources and methods of selection of participants. Describe methods of follow-up  Included in Material and Methods. Page 6 and 7. |
|  |  | (*b*) For matched studies, give matching criteria and number of exposed and unexposed  Not applicable. |
| Variables | 7 | Clearly define all outcomes, exposures, predictors, potential confounders, and effect modifiers. Give diagnostic criteria, if applicable  Included in Material and Methods. Page 8. |
| Data sources/ measurement | 8* | For each variable of interest, give sources of data and details of methods of assessment (measurement). Describe comparability of assessment methods if there is more than one group  Included in Material and Methods. Page 8 and 9. |
| Bias | 9 | Describe any efforts to address potential sources of bias  Addresses the limitations in the final paragraph of the Discussion section. Page 15. |
| Study size | 10 | Explain how the study size was arrived at  The sample size is detailed in the Statistical Analysis and Sample Size Calculation subsection within the Materials and Methods section. Page 9. |
| Quantitative variables | 11 | Explain how quantitative variables were handled in the analyses. If applicable, describe which groupings were chosen and why  Included in Materials and Methods. Page 9 and 10. |
| Statistical methods | 12 | (*a*) Describe all statistical methods, including those used to control for confounding  Included in the Statistical Analysis and Sample Size Calculation subsection within the Materials and Methods section. Page 9 and 10. |
|  |  | (*b*) Describe any methods used to examine subgroups and interactions  Included in the Statistical Analysis and Sample Size Calculation subsection within the Materials and Methods section. Page 10. |
|  |  | (*c*) Explain how missing data were addressed  Not applicable. |
|  |  | (*d*) If applicable, explain how loss to follow-up was addressed  Not applicable. |
|  |  | (*e*) Describe any sensitivity analyses  Not applicable. |
| Results | | |
| Participants | 13* | (a) Report numbers of individuals at each stage of study—e.g. numbers potentially eligible, examined for eligibility, confirmed eligible, included in the study, completing follow-up, and analyzed  Included in the Results. Page 10. |
|  |  | (b) Give reasons for non-participation at each stage  Not applicable. |
|  |  | (c) Consider use of a flow diagram  Included in the Results. Page 10. |
| Descriptive data | 14* | (a) Give characteristics of study participants (e.g. demographic, clinical, social) and information on exposures and potential confounders  Included in the Results. Page 10 and 11. |
|  |  | (b) Indicate number of participants with missing data for each variable of interest  Provided in the Results, and in the flow diagram (Figure 1). Page 10. |
|  |  | (c) Summarise follow-up time (e.g., average and total amount)  Included in the Results. Page 12. |
| Outcome data | 15* | Report numbers of outcome events or summary measures over time  Included in the Results. Page 11 and 12. |
| Main results | 16 | (*a*) Give unadjusted estimates and, if applicable, confounder-adjusted estimates and their precision (e.g., 95% confidence interval). Make clear which confounders were adjusted for and why they were included  Included in the Results. |
|  |  | (*b*) Report category boundaries when continuous variables were categorized  Not applicable. |
|  |  | (*c*) If relevant, consider translating estimates of relative risk into absolute risk for a meaningful time period  Not applicable. |
| Other analyses | 17 | Report other analyses done—e.g. analyses of subgroups and interactions, and sensitivity analyses  Included in the Results. |
| Discussion | | |
| Key results | 18 | Summarise key results with reference to study objectives  Included in the Discussion. Page 12 and 13. |
| Limitations | 19 | Discuss limitations of the study, taking into account sources of potential bias or imprecision. Discuss both direction and magnitude of any potential bias  Included in the Discussion. Page 15. |
| Interpretation | 20 | Give a cautious overall interpretation of results considering objectives, limitations, multiplicity of analyses, results from similar studies, and other relevant evidence  Included in the Discussion. |
| Generalisability | 21 | Discuss the generalisability (external validity) of the study results  Included in the Discussion. Page 15. |
| Other information | | |
| Funding | 22 | Give the source of funding and the role of the funders for the present study and, if applicable, for the original study on which the present article is based  Included in the last page. Page 16 |
